# Use of the viral 2A peptide for bicistronic expression in transgenic mice

**DOI:** 10.1186/1741-7007-6-40

**Published:** 2008-09-15

**Authors:** Georgios Trichas, Jo Begbie, Shankar Srinivas

**Affiliations:** 1 Department of Physiology Anatomy and Genetics, University of Oxford, South Parks Road, Oxford OX1 3QX, UK

## Abstract

**Background:**

Transgenic animals are widely used in biomedical research and biotechnology. Multicistronic constructs, in which several proteins are encoded by a single messenger RNA, are commonly used in genetically engineered animals. This is currently done by using an internal ribosomal entry site to separate the different coding regions. 2A peptides result in the co-translational 'cleavage' of proteins and are an attractive alternative to the internal ribosomal entry site. They are more reliable than the internal ribosomal entry site and lead to expression of multiple cistrons at equimolar levels. They work in a wide variety of eukaryotic cells, but to date have not been demonstrated to function in transgenic mice in an inheritable manner.

**Results:**

To test 2A function in transgenic mice and uncover any possible toxicity of widespread expression of the 2A peptide, we made a bicistronic reporter construct containing the coding sequence for a membrane localised red fluorescent protein (Myr-TdTomato) and a nuclear localised green fluorescent protein (H2B-GFP), separated by a 2A sequence. When this reporter is transfected into HeLa cells, the two fluorescent proteins correctly localise to mutually exclusive cellular compartments, demonstrating that the bicistronic construct is a reliable readout of 2A function. The two fluorescent proteins also correctly localise when the reporter is electroporated into chick neural tube cells. We made two independent transgenic mouse lines that express the bicistronic reporter ubiquitously. For both lines, transgenic mice are born in Mendelian frequencies and are found to be healthy and fertile. Myr-TdTomato and H2B-GFP segregate to mutually exclusive cellular compartments in all tissues examined from a broad range of developmental stages, ranging from embryo to adult. One transgenic line shows X-linked inheritance of the transgene and mosaic expression in females but uniform expression in males, indicating that the transgene has integrated into the X chromosome in this line.

**Conclusion:**

The 2A peptide efficiently mediates co-translational cleavage in transgenic mice in which it has been inherited through the germ-line. Mice expressing it ubiquitously throughout development and into adulthood appear normal. It is therefore a viable tool for use in genetically engineered mice and represents a superior alternative to the widely used internal ribosomal entry site.

## Background

Transgenic animals play a central part in biomedical research and biotechnology. The ability to genetically modify mice, either through random genomic integration by pronuclear injection or through targeted modification by homologous recombination in embryonic stem cells, has transformed the field of mouse molecular genetics, making it now a relatively routine procedure to intervene genetically to study a biological process or address the function of a gene. Such transgenic approaches have also been successfully applied to larger mammals for the production of livestock that synthesise human proteins or possess specific growth characteristics.

It is often advantageous to make transgenic animals that express multiple genes under the control of a single promoter. This allows the expression of different proteins in a co-ordinated manner while simplifying the genetics and reducing the cost, since multiple transgenic lines do not need to be bred together. Currently, the favoured method for achieving this is to use an internal ribosomal entry site (IRES). The IRES was first identified in the encephalomyocarditis virus, a member of the genus *Cardiovirus*, which belong to the family of small RNA viruses called *Picornaviridae *[1]. The IRES allows multiple proteins to be made from a single mRNA transcript as ribosomes bind to the IRES in a 5'-cap-independent manner and initiate translation. They can therefore be used to engineer multicistronic transgenes, in which several open reading frames are encoded on a single transcript, separated by IRES sequences. IRES sequences are also commonly used in gene targeting experiments in mice to express a foreign gene under the control of an endogenous promoter. In these cases, the 'knocked-in' foreign gene is preceded by an IRES, allowing it to be produced as a separate protein, rather than as a fusion with the protein encoded by the endogenous gene. In addition to being used in transgenic mice, IRES sequences are very widely used in tissue-culture cell transfections, as a means of ensuring cells express the transfected gene by using constructs in which the gene of interest is followed by an IRES and a selectable drug resistance gene or fluorescent marker.

IRES sequences, however, have several limitations: the expression of the downstream cistron is sensitive to its specific positioning after the IRES and hence the IRES can be unreliable; the downstream coding sequence is often translated at much lower levels than the upstream sequence; they are not small (approximately 600 base pairs), adding to the size of the transgene; they do not provide equivalent levels of expression of the genes separated by IRES elements. Although efforts continue to be made to address the first of the above issues [2], the other problems are harder to address.

Recently, the use of the 2A peptide in multicistronic constructs has emerged as an attractive alternative to the IRES. Like the IRES, the 2A peptide was identified among picornaviruses but in a different sub-group, the *Aphthoviruses*, a typical example of which is the *Foot-and-mouth disease virus *[3]. 2A-like sequences have since been found in other *Picornaviridae *like the *Equine rhinitis A virus*, as well as unrelated viruses such as the *Porcine teschovirus-1 *and the insect *Thosea asigna virus *(TaV) [4]. In such viruses, multiple proteins are derived from a large polyprotein encoded by a single open reading frame. The 2A peptide mediates the co-translational cleavage of this polyprotein at a single site that forms the junction between the virus capsid and replication polyprotein domains.

The 2A sequences are relatively short peptides (of the order of 20 amino acids long, depending on the virus of origin) containing the consensus motif Asp-Val/Ile-Glu-X-Asn-Pro-*Gly*-*Pro*. They were originally thought to mediate the autocatalytic proteolysis of the large polyprotein, but are now understood to act co-translationally, by preventing the formation of a normal peptide bond between the glycine and last proline, resulting in the ribosome skipping to the next codon [5], and the nascent peptide cleaving between the Gly and Pro. After cleavage, the short 2A peptide remains fused to the C-terminus of the 'upstream' protein, while the proline is added to the N-terminus of the 'downstream' protein. It has recently been shown that translation termination release factors eRF1 and eRF3 play an important role in the function of the 2A peptide [6]. Due to its mode of action, the 2A peptide is described as a 'cis-acting hydrolase element' (CHYSEL) [7].

The 2A peptide was demonstrated to efficiently mediate the co-translational cleavage of artificial polypeptides by inserting it between the coding sequences of two reporter genes [8]. Subsequently, it has been shown to function in cells from a wide variety of eukaryotes, ranging from yeast to plants to insects to mammals [7]. The 2A peptide was first shown to work *in vivo *in the rat brain, using recombinant adeno-associated viruses containing 2A dependent vectors [9]. More recently, a striking demonstration of its potential has been to use it to reconstitute the expression of the multisubunit T-cell receptor:CD3 complex in bone marrow-derived stem cells transduced with retroviral vectors carrying up to four CD3 genes separated by 2A sequences [10,11]. When transplanted back into CD3-deficient mice, these transduced stem cells gave rise to T cells, demonstrating 2A function. The 2A peptide was also shown to function in derivatives of the transduced stem cells (in the spleen, thymus and blood) and to result in stoichiometric co-expression of multiple proteins [10]. The many advantages of the 2A peptide in biotechnology have been reviewed by de Felipe and colleagues [12].

Although the 2A peptide has been shown to function in a wide variety of cell types and in specific tissues *in vivo*, it has to date not been demonstrated to function in a permanent transgenic mouse line after passage through the germ-line, to test for possible silencing of 2A-containing transgenes. Moreover, it is not known if there might be possible toxic side-effects of the 2A peptide upon widespread continuous expression at various stages of development. Therefore, we have made transgenic mouse lines that express a 2A-containing construct essentially ubiquitously throughout development and adulthood. We report that such mice appear normal and healthy, and that the 2A peptide functions in all tissues at all stages examined in such mice.

## Results and discussion

### Constructing a fluorescent reporter of 2A function

We designed a strategy to enable us to assess visually 2A function using fluorescent proteins localised to two distinct cellular compartments. We linked a membrane localised TdTomato gene (*Myr-TdTomato*) to a nuclear localised enhanced green fluorescent protein (EGFP) gene (*H2B-GFP*) using the 2A sequence of the TaV that has previously been used to efficiently mediate the cleavage of polyproteins [10]. TdTomato fluoresces red and EGFP fluoresces green, allowing one to distinguish them unambiguously.

**Figure 1:**
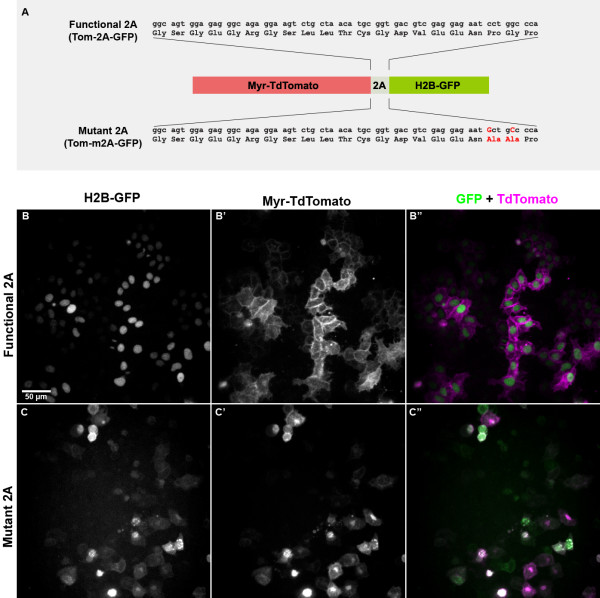
**Testing 2A constructs in HeLa cells**. (A) Constructs Tom-2A-GFP and Tom-m2A-GFP used to test the function of the 2A linker. A membrane localised red fluorescent protein (Myr-TdTomato) linked to a nuclear localised green fluorescent protein (H2B-GFP) by either a functional 2A linker (top), or by a non-functional m2A linker (bottom). The specific nucleotide and amino acid changes made in Tom-m2A-GFP are shown in red. (B-B") HeLa cells lipofected with Tom-2A-GFP. Fluorescent cells show segregation of the TdTomato and enhanced green fluorescent protein (EGFP) to mutually exclusive cellular compartments. (C-C") HeLa cells lipofected with Tom-m2A-GFP as a negative control. The EGFP and TdTomato are no longer segregated properly, consistent with them remaining linked together as a single fusion protein due to the mutations in the 2A linker. (B, C) EGFP fluorescence. (B', C') TdTomato fluorescence. (B", C") Merge of EGFP (green) and TdTomato (magenta) fluorescence. Diagram in (A) not to scale. The scale bar in (B) represents 50 μm and applies to panels B through C".

As a negative control, we made an identical fusion construct, but with two nucleotide changes that abolish 2A function [10]. These two versions of the construct were called Tom-2A-GFP and Tom-m2A-GFP, respectively (Figure 1A).

We tested both constructs by expressing them in HeLa cells under the control of a CMV-IE promoter. In cells lipofected with Tom-2A-GFP, EGFP was detected exclusively in the nucleus while TdTomato was detected predominantly in the plasma membrane, and at lower levels within the cell, presumably in membrane organelles or the cytoplasm (Figure 1B–B"). In contrast, in cells lipofected with Tom-m2A-GFP (the mutant construct), EGFP was detected in the plasma membrane and cytoplasm in addition to the nucleus (Figure 1C). Similarly, TdTomato was detected in both the plasma membrane and nucleus (Figure 1C'), demonstrating that the mutually exclusive localisation of TdTomato and EGFP seen with the Tom-2A-GFP construct is a reliable readout of 2A function.

**Figure 2:**
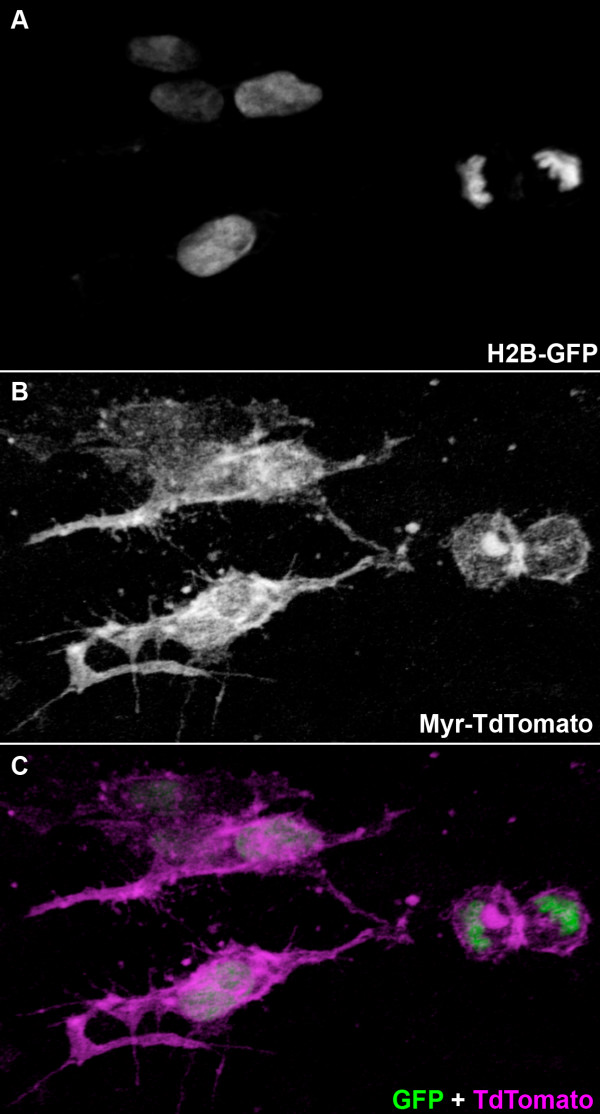
**Testing the Tom-2A-GFP construct in chick embryos**. (A-C) Three-dimensional volume rendering of a confocal image stack of embryonic chick neural crest cells electroporated with the Tom-2A-GFP construct under the control of a CMV enhancer. The enhanced green fluorescent protein (EGFP) and TdTomato segregate to the nucleus and plasma membrane respectively, indicating that the 2A peptide functions in chick cells. (A) EGFP fluorescence. (B) TdTomato fluorescence. (C) Merge of EGFP (green) and TdTomato (magenta) fluorescence. Scale indicators are rendered in perspective along with the confocal image data and do not translate reliably to a two-dimensional image, therefore, no scale bar is depicted. As an approximate indicator, the two cells at the right of the image that have just divided are, together, between 15 and 20 μm long.

In order to confirm that the Tom-2A-GFP construct worked not only in cultured cells but also in endogenous vertebrate cells, we electroporated it into the neural tube of chick embryos, labelling delaminating neural crest cells. In confirmation of the results with the HeLa cells, EGFP localised to the nucleus and TdTomato to the plasma membrane in electroporated cells, indicating that the 2A peptide functions in chick cells (Figure 2).

### Production of mice transgenic for Tom-2A-GFP

Having established that the Tom-2A-GFP construct is a reliable indicator of 2A function, we made a transgene in which it is expressed under the control of the CAG promoter. The CAG promoter drives expression widely in the mouse [13], allowing us to assess 2A function and possible deleterious effects in a wide range of tissue types and developmental stages. Transgenic mice were made by pronuclear injection and two transgenic founders called CAG-TAG1 and CAG-TAG2 were obtained. Both founders were bred to establish permanent lines.

The transgene is expressed in both lines and is inherited in a Mendelian manner, suggesting that it does not cause embryonic or perinatal lethality. Transgenic mice of both lines are indistinguishable from their non-transgenic littermates, suggesting that the widespread expression of the 2A peptide has no deleterious effects on the health of transgenic mice in either of these two independent lines. CAG-TAG2 shows an X-linked mode of inheritance, indicating the transgene integrated into the X chromosome. Both lines were further characterised, as described below.

### Function of the 2A peptide in transgenic embryos

**Figure 3:**
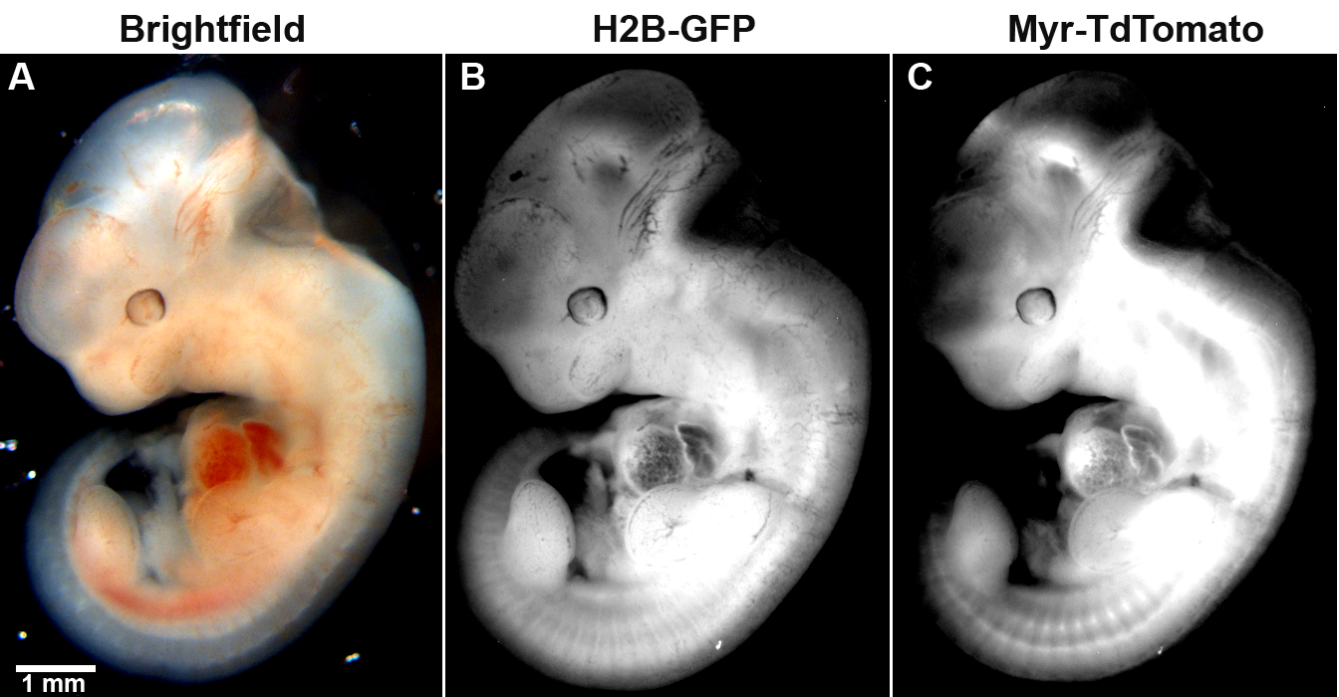
**Widespread expression of Tom-2A-GFP in transgenic embryos**. An E11.5 mouse embryo transgenic for Tom-2A-GFP under the control of a CAG promoter is shown. (A) Brightfield image, (B) Enhanced green fluorescent protein (EGFP) fluorescence and (C) TdTomato fluorescence. EGFP and TdTomato can be seen to be expressed throughout the embryo. Fluorescence intensity of both EGFP and TdTomato is higher in regions of relatively greater tissue density. The scale bar in (A) represents 1 mm and applies to all panels.

To assess 2A function after being inherited through the germ-line, we first looked in heterozygous embryos that were two generations removed from the original founder transgenic mouse. Embryos from both transgenic lines showed widespread expression of the Tom-2A-GFP, as expected from the CAG promoter used (Figure 3). Robust levels of fluorescence were detected in both living as well as fixed samples from both transgenic lines.

**Figure 4:**
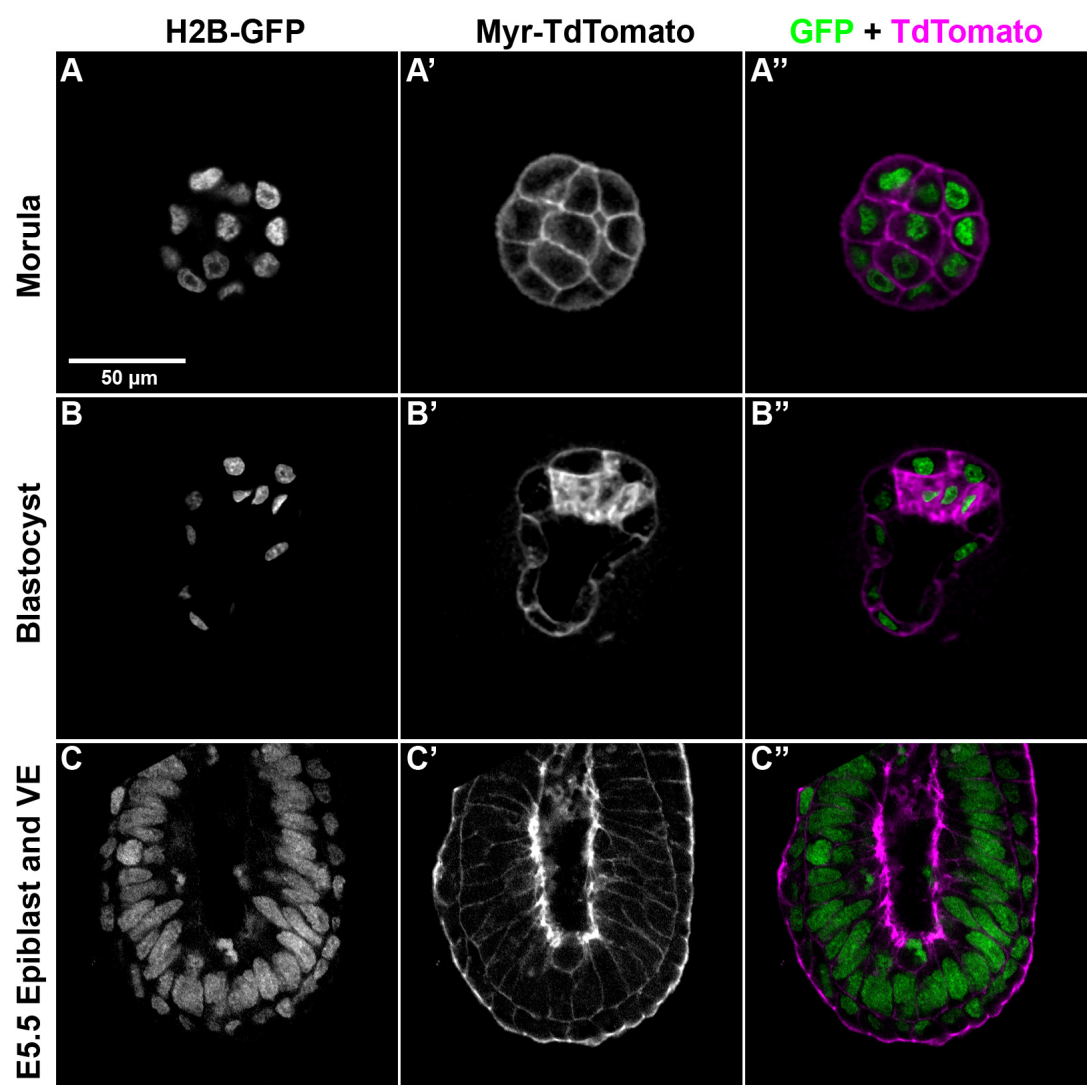
**Function of 2A peptide in pregastrulation embryos**. (A-A") E2.5, (B-B") E4.0 and (C-C") E5.5 embryos expressing Tom-2A-GFP ubiquitously under the control of a CAG promoter. The embryos shown represent the F2 generation with respect to the founder F0 transgenic mouse. In all the embryos shown, the transgene was inherited from a heterozygous father. Cells in both embryonic as well as extra-embryonic tissues show segregation of TdTomato and enhanced green fluorescent protein (EGFP) to mutually exclusive cellular compartments, indicating 2A function in both lineages. (A, B, C) EGFP fluorescence. (A', B', C') TdTomato fluorescence. (A", B", C") Merge of EGFP (green) and TdTomato (magenta) fluorescence. The scale bar in (A) represents 50 μm and applies to all panels.

In both lines, at all pregastrulation stages, EGFP is localised exclusively to the nucleus, while TdTomato is present predominantly in the plasma membrane (Figure 4 and data not shown for CAG-TAG2 embryos). Fluorescence is first detected at the two-cell stage when the transgene is inherited paternally. Embryos that inherit the transgene maternally show fluorescence as zygotes, due to the inheritance of maternal RNA (data not shown).

Interestingly, at the blastocyst stage, TdTomato is expressed widely in the cytoplasm of cells of the inner cell mass (ICM), even though in adjacent trophectoderm cells, it is seen mostly in the plasma membrane. This might reflect differences between trophectoderm and ICM cells in the processing of myristoylated proteins. TdTomato is never detected in the nucleus and EGFP never in the cytoplasm or plasma membrane, confirming that it is being cleaved from the H2B-GFP in both ICM and trophectoderm cells.

**Figure 5:**
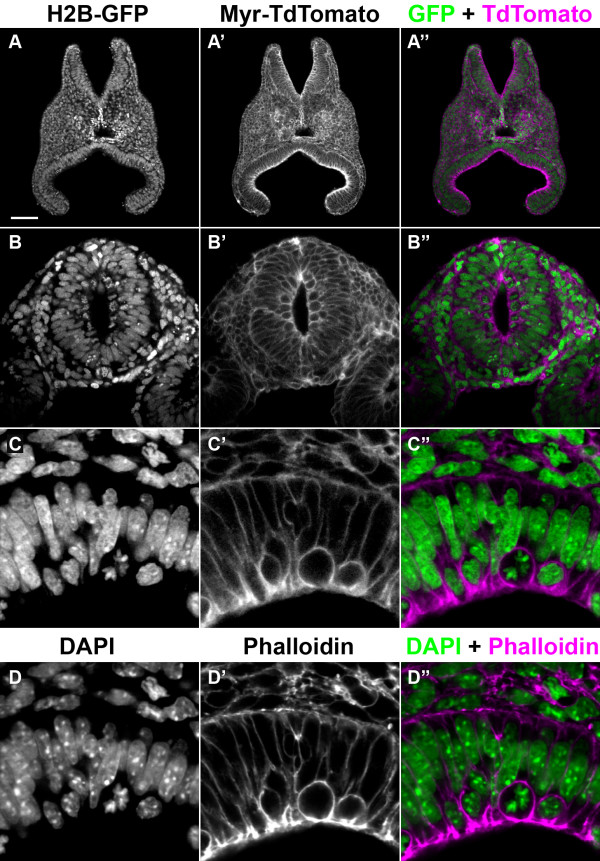
**Function of 2A peptide in E8.5 embryos**. (A-A") Transverse section through an E8.5 transgenic embryo. (B-B") Transverse section through the neural tube of an E8.5 transgenic embryo. Note the 'ring' of dividing cells lining the inside of the neural tube. (C-D") High magnification view of neural tube cells expressing Tom-2A-GFP, stained with 4',6-diamidino-2-phenylindole (DAPI) (nucleus) and phalloidin (sub-cortical actin marking plasma membrane), confirming that enhanced green fluorescent protein (EGFP) and TdTomato are expressed in mutually exclusive compartments, in the nucleus and plasma membrane respectively. (A, B, C) EGFP fluorescence. (A', B', C') TdTomato fluorescence. (A", B", C") Merge of EGFP (green) and TdTomato (magenta) fluorescence. (D) DAPI fluorescence. (D') Atto647N-phalloidin fluorescence. (D") Merge of DAPI (green) and Atto647N (magenta). The scale bar in (A) represents 63 μm in (A-A"), 31 μm in (B-B") and 10 μm in (C-D").

Mutually exclusive localisation of EGFP and TdTomato to the nucleus and membrane was also observed in tissues derived from all three germ-layers at later embryonic stages, such as E8.5 (Figure 5), E11.5 and E18.5 (data not shown). In order to verify independently that the EGFP was indeed localised to the nucleus, we stained tissue sections with the nuclear dye 4',6-diamidino-2-phenylindole (DAPI), which confirmed the nuclear localisation of EGFP (Figure 5C and 5D). Similarly, a phalloidin stain (which labels sub-cortical actin filaments), verified the localisation of TdTomato to the plasma membrane (Figure 5C' and 5D'). We, however, cannot exclude the possibility that extremely small amounts of un-cleaved protein, below the detection capability of our confocal microscope, are produced and incorrectly localised.

### Function of the 2A peptide in adult transgenic mice

**Figure 6:**
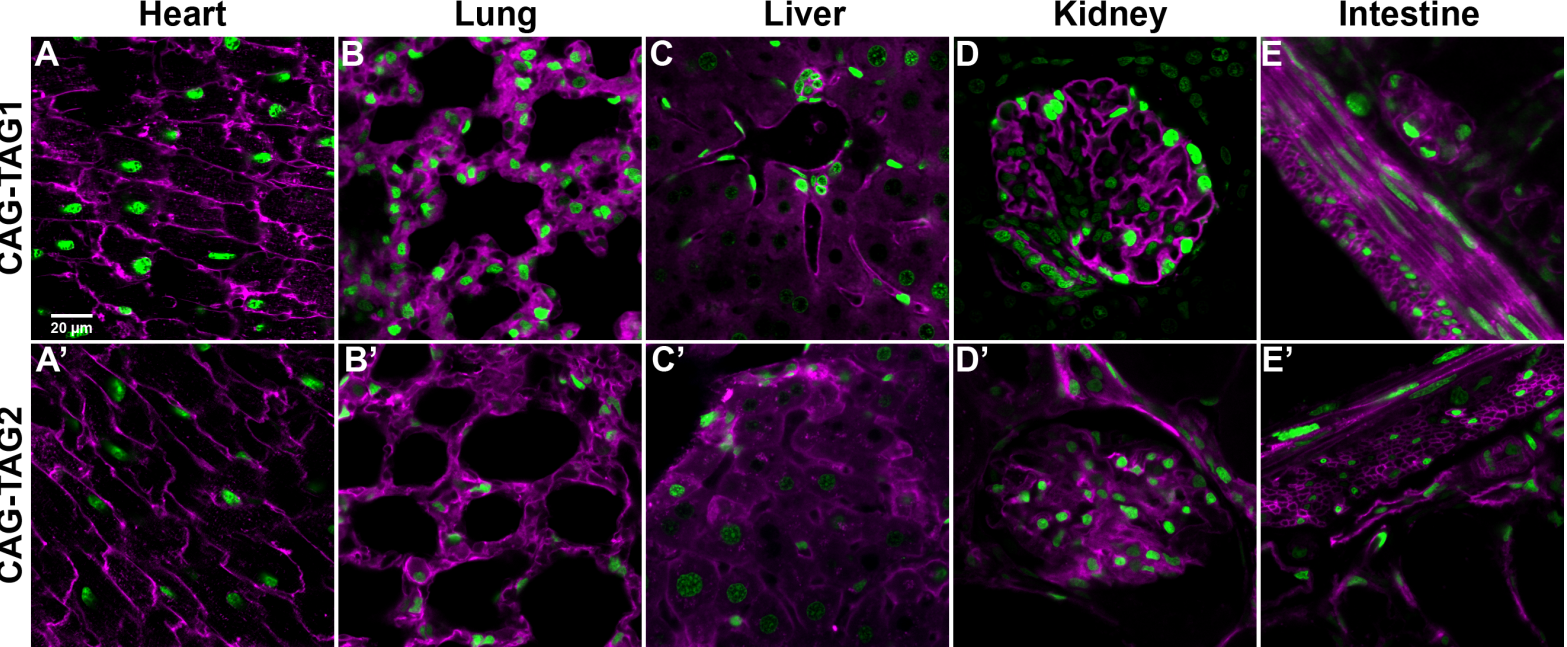
**Function of 2A peptide in various adult tissues**. Sections through the heart (A, A'), lung (B, B'), liver (C, C'), kidney (D, D') and intestine (E, E') of adult CAG-TAG1 and CAG-TAG2 transgenic mice. The enhanced green fluorescent protein signal is depicted in green and TdTomato signal is depicted in magenta. No overlap of the H2B-GFP and Myr-TdTomato signal is seen, demonstrating efficient functioning of the 2A peptide. The scale bar in (A) represents 20 μm and is applicable to all panels.

We next assessed 2A function in various tissues from male adult heterozygous transgenic mice that were three generations removed from the original founder mouse. H2B-GFP and Myr-TdTomato were localised to mutually exclusive cellular compartments in all the tissues examined: the brain, heart, lung, liver, kidney, ileum, colon and adrenal (Figure 6 and data not shown) for both transgenic lines. In cells of the cardiac muscle, intestine and kidney, TdTomato was found to localise to the plasma membrane. However, in cells of the lungs and liver, it was less distinctly localised to the plasma membrane (Figure 6B, B', C and 6C'), as previously observed with cells of the embryonic ICM. Again, TdTomato and EGFP were never found to co-localise in the two independent transgenic lines, indicating efficient cleavage of the 2A peptide.

### Mosaic transgene expression in female CAG-TAG2 mice

**Figure 7:**
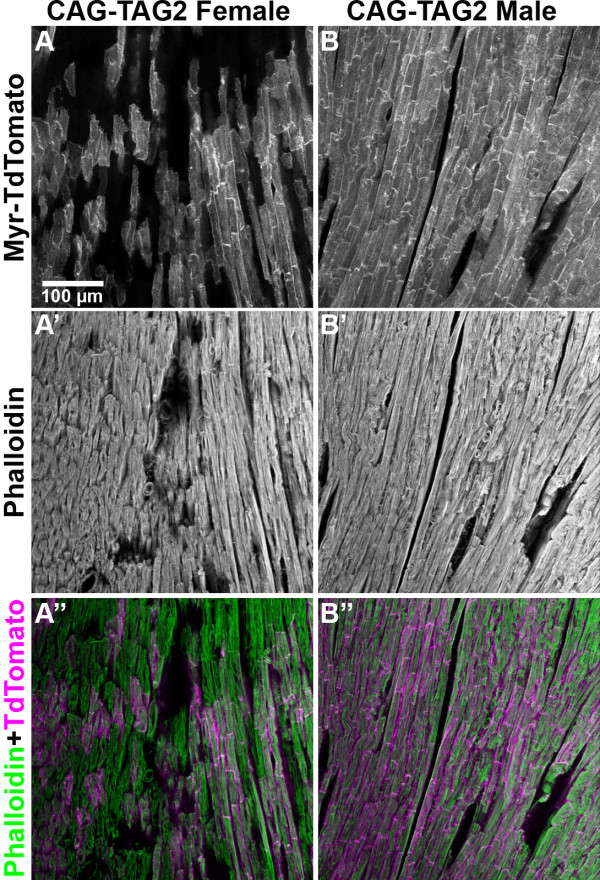
**Mosaic expression of CAG-TAG2 due to X inactivation**. Sections through the heart of an adult female (A, A', and A") and male (B, B' and B") CAG-TAG2 mouse are shown. (A, B) Td-Tomato fluorescence showing expression of the transgene. (A', B') Atto647N-phalloidin fluorescence used as a 'counter-stain' to visualise all the tissue in the field of view. (A", B") Merge of TdTomato (red) and phalloidin (green) fluorescence, illustrating the mosaic expression of the CAG-TAG transgene in females due to X inactivation, but uniform expression in males. The scale bar in (A) represents 100 μm and is applicable to all panels.

The CAG-TAG2 transgene shows an X-linked mode of inheritance, indicating that the transgene integrated in the X chromosome. Consistent with this, females of the CAG-TAG2 line show mosaic expression due to X inactivation (Figure 7), while males show uniform expression (as in both genders of the CAG-TAG1 line). Both genders, however, show the correct subcellular localisation of TdTomato and EGFP, demonstrating that the 2A peptide functions in both transgenic lines.

### Expression levels of the CAG-TAG transgene is constant across generations

**Figure 8:**
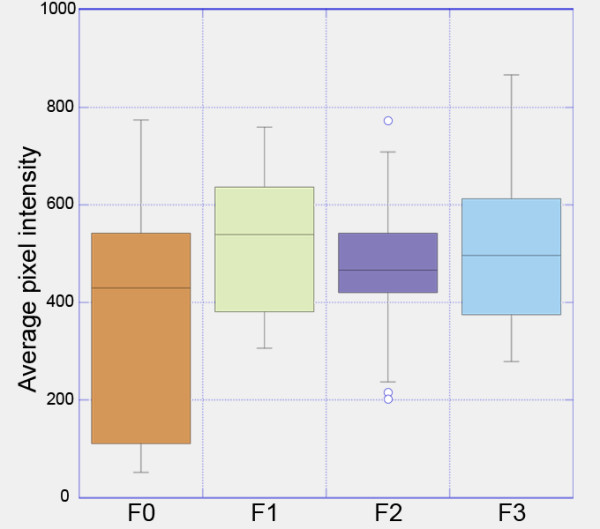
**Expression levels of CAG-TAG1 transgene across four generations**. TdTomato fluorescence intensity was used as a measure of transgene expression levels. TdTomato fluorescence was quantified in cardiac tissue from the CAG-TAG1 male founder (F0), and from his F1, F2, and F3 male adult offspring. The plot represents the distribution of average TdTomato fluorescence intensity per pixel for 10 different fields of view for each sample. F1, F2 and F3 mice express the transgene at equivalent levels. They do not show a significant difference in expression levels (Student t-test, *P *> 0.1 for all three combinations of pair-wise comparisons of the three samples). The founder has a broader distribution of average intensity per pixel and shows a lower minimum value than the others because he was mosaic for the transgene, and some of the fields of view measured had regions that did not express the transgene, causing a lowering of the average pixel intensity.

In order to determine if there is any variability of expression levels of the CAG-TAG transgene across individuals or diminution of expression across generations, we quantified the fluorescence intensity of TdTomato as an estimate of its expression level. We compared expression in cardiac tissue from the CAG-TAG1 founder (F0), F1, F2 and F3 adult male mice (Figure 8). Transgene expression levels were very similar in the F1, F2 and F3 individuals, showing no statistically significant difference. This suggests the expression levels of the CAG-TAG1 transgene does not vary significantly across individuals, and more importantly, does not show a reduction with repeated passage through the germ-line.

The founder mouse showed a lower median expression level compared with his offspring. This is consistent with the founder being mosaic for the transgene, which is not uncommon in transgenic founders. It is also consistent with the observation that the cardiac tissue from the founder showed mosaic transgene expression (data not shown). It is important to note that those regions of founder tissue that did express the transgene appear to have done so at levels comparable with that found in the offspring, as illustrated by the overlap of the upper quartile of fluorescence intensities observed in the founder, with that observed in his offspring (Figure 8).

## Conclusion

Our results indicated that the 2A peptide does not produce cytotoxic effects in transgenic mice that express it in all their cells throughout development and adulthood. They also indicate that the 2A peptide functions in transgenic mice after several generations of passage through the germ-line and transgenes encoding the 2A peptide do not show attenuation in expression levels across generations. Our results also confirmed that the 2A peptide mediates co-translational 'cleavage' in a wide range of embryonic and adult tissues.

Together, this establishes the 2A peptide as a viable and, being more reliable and easier to use, a superior alternative to the IRES in mouse transgenesis. It fulfils all the functions IRES sequences are currently used for: multicistronic expression in transgenic animals and cell culture, multicistronic expression using viral vectors in entire animals, expression of exogenous coding sequences inserted by targeted recombination into endogenous loci, etc. In addition, it also provides the advantage of reliable and stoichiometric levels of expression, making it particularly useful when the relative level of expression of two or more transgenic proteins is important.

Finally, the transgenic mice reported here are useful in their own right for visualising cell membranes and nuclei in living and fixed tissue. Owing to the widespread expression of the CAG-TAG transgene, the mice are likely to be of use in a broad range of disciplines. In conjunction with time-lapse microscopy, they represent a powerful tool for following various cellular parameters such as shape, volume, movement and division rates in cultured cells, organs or entire embryos. The fortuitous insertion of the transgene in the X chromosome in one of the transgenic lines makes that line particularly useful in monitoring X chromosome inactivation.

## Methods

### Plasmids and constructs

pCS-mCherry (a gift from Sean Megason) contains two tandem copies of a myristoylation signal in frame with *mCherry*, which targets mCherry to the membrane. We replaced the *mCherry *in this plasmid with *TdTomato *cloned from plasmid pSET-B-tdTomato (a gift from Roger Tsien), because of its increased brightness and photostability [14]. While replacing *mCherry *with *TdTomato*, we destroyed a Kozak consensus sequence between the myristoylation sequence and the initiation codon of *mCherry*, in order to increase the probability that translation would initiate at another Kozak site upstream of the two myristoylation sequences, thereby reducing possible cytoplasmic 'background' localisation.

*TdTomato *was polymerase chain reaction-amplified using one forward primer and two different reverse primers, one encoding the normal 2A peptide and the other the non-cleavable mutant 2A peptide (referred to here as m2A) [10]. Both reverse primers omitted the *TdTomato *stop codon, so that translation could continue into the 2A sequence and beyond. To facilitate subsequent cloning, the forward primer incorporated an AgeI site, and the two lower primers incorporated a BglII and SnabI site. pCS-mCherry was digested with AgeI and SnabI to remove mCherry and replaced with amplified *TdTomato-2A *or *TdTomato-m2A *digested with the same enzymes. The resulting plasmids were called pCS-TdTomato-2A and pCS-TdTomato-m2A.

Next *H2B-GFP *was cut out of plasmid pCAG-H2B-GFP-STP (a gift from Shahragim Tajbakhsh) with BglII and NotI and cloned in frame downstream of *TdTomato-2A *and *TdTomato-m2A*. The resulting plasmids called pCS2-TdTomato-2A-GFP and pCS2-TdTomato-m2A-GFP were tested by lipofection in HeLa cells.

To make the transgene construct for pronuclear injection, *TdTomato-2A-GFP *was excised with XhoI (made blunt with Klenow) and NotI and inserted downstream of the CAG promoter of pCAG-H2B-GFP-STP cut with EcoRV and NotI. A linear fragment for injection containing CAG-TdTomato-2A-GFP was excised with XhoI and NotI.

### Primers

Forward primer:

5' GCA CCG GTC GCC TCC ATG GTG AGC AAG GGC GAG GAG GTC ATC 3'

2A reverse primer:

5' C CTA CGT AAG ATC TCC *TGG GCC AGG ATT CTC CTC GAC GTC ACC GCA TGT TAG CAG ACT TCC TCT GCC CTC TCC ACT GCC *CTT GTA CAG CTC GTC CAT GCC GTA CAG 3'

m2A reverse primer:

5' C CTA CGT AAG ATC TCC *TGG GGC AGC ATT CTC CTC GAC GTC ACC GCA TGT TAG CAG ACT TCC TCT GCC CTC TCC ACT GCC *CTT GTA CAG CTC GTC CAT GCC GTA CAG 3'

Underlined regions of the reverse primers encode the 2A (or m2A) peptide.

### Lipofection

pCS2-TdTomato-2A-GFP and pCS2-TdTomato-m2A-GFP were lipofected into HeLa cells using Lipofectamine-2000 from Invitrogen according to the manufacturer's protocol; 50 ng of plasmid DNA was used per well of a 24-well plate.

### Chick electroporation

pCS2-TdTomato-2A-GFP at 2 μg/μl was electroporated *in ovo *into the neural tube of chick embryos at stage HH 12 using four 5-ms pulses of 10 V. Electroporated embryos were incubated for 24 hours at 38°C, then dissected, fixed and scanned using a confocal microscope.

### Transgenic mice and breeding

For pronuclear injection into mouse zygotes, the transgene was excised by restriction enzyme digest and gel purified using the Qiagen gel purification kit according to the manufacturer's recommendations, except that the DNA was eluted directly with injection buffer (10 mM Tris-Cl, 0.1 mM ethylenediaminetetraacetic acid). The transgene DNA was diluted to a concentration of 3.0 ng/μl and used for pronuclear injection into [B6xCBA]F2 mouse zygotes as described [15]. Potential transgenic mice were screened by examining tail-tip biopsies under a fluorescence stereomicroscope (Zeiss StereoLumar), and transgenic mice were bred to [B6xCBA]F1 mice (Harlan) to establish permanent lines.

### Embryo and tissue collection, sectioning and staining

To produce staged embryos, transgenic males were crossed to wild type CD1 females (Charles River). All mice were maintained on a 12 hour light, 12 hour dark cycle. Noon on the day of finding a vaginal plug was designated 0.5 *dpc*. Embryos of various stages and tissues from adult mice were dissected in M2 medium and fixed in 4% paraformaldehyde in phosphate buffered saline (PBS) overnight. Samples were then washed in PBT (PBS + 0.1% Triton-X100) several times and then either mounted whole in DAPI-Vectashield (Vector Laboratories, H-1200) or processed for cryosectioning. Samples for sectioning were incubated in 30% sucrose in PBS at 4°C overnight. They were then embedded in a solution of 15% sucrose and 7.5% gelatin in PBS and frozen in isopentane maintained at -55°C. Frozen sections were cut at 30 μm thickness at -25°C. Sections were washed three times for 5 minutes each in PBT, incubated for 30 minutes with Atto647N-phalloidin (Sigma – 65906) diluted 1:200 in PBT, washed five times for 5 minutes each in PBT and then mounted in DAPI-Vectashield.

### Image data colletion

The lipofected cells in Figure 1 were imaged on an Improvision spinning disc microscope with a Yokagawa CSU10 head and a Hamamatsu EM-CCD camera mounted on a Zeiss inverted microscope, using a ×40/0.6 NA air objective. EGFP was excited at 492 nm and TdTomato at 561 nm.

Fixed samples were imaged on Zeiss LSM510 META and Zeiss LSM 710 confocal microscopes using ×10/0.45 NA, ×20/0.75 NA, ×40/1.3 NA or ×63/1.4 NA lenses as appropriate. EGFP was excited at 488 nm; TdTomato was excited at 543 nm; DAPI was excited at 405 nm; and Atto647N-phalloidin was excited at 633 nm. All image analysis and three-dimensional rendering was done using Volocity (Improvision, UK). Figures were prepared using Photoshop CS2 (Adobe Inc.).

### Fluorescence quantification

For TdTomato fluorescence intensity quantification, all samples were collected at the same time and processed identically. Frozen sections of cardiac muscle tissue were scanned as above, at 12 bit depth under non-saturating conditions. The average intensity per pixel was measured for 10 random fields of view for each sample, using ImageJ. Keliadegraph was used to plot the distribution of intensities and to determine the significance of any variability between the samples using the Students t-test.

## List of abbreviations

DAPI: 4',6-diamidino-2-phenylindole; EGFP: enhanced green fluorescent protein; ICM: inner cell mass; IRES: internal ribosomal entry site; PBS: phosphate buffered saline; PBT: PBS + 0.1% Triton-X100; TaV: *Thosea asigna virus*

## Authors' contributions

GT made all the constructs described; carried out the pronuclear injections to make transgenic mice; carried out the lipofections; carried out the chick electroporations under the guidance of JB; collected most of the embryos; sectioned and imaged all the samples. JB made available her chick embryo and electroporation expertise, along with equipment and necessary materials. SS conceived and directed the project; helped with the pronuclear injections and embryo transfers; prepared the figures and wrote the majority of the manuscript.
